# RING Finger Protein 11 Targets TBK1/IKKi Kinases to Inhibit Antiviral Signaling

**DOI:** 10.1371/journal.pone.0053717

**Published:** 2013-01-07

**Authors:** Soratree Charoenthongtrakul, Linlin Gao, Kislay Parvatiyar, David Lee, Edward W. Harhaj

**Affiliations:** 1 Department of Microbiology and Immunology, The University of Miami, Miller School of Medicine, Miami, Florida, United States of America; 2 Graduate Program in Cancer Biology, Sylvester Comprehensive Cancer Center, The University of Miami, Miller School of Medicine, Miami, Florida, United States of America; 3 Department of Oncology, Johns Hopkins School of Medicine, Sidney Kimmel Comprehensive Cancer Center, Baltimore, Maryland, United States of America; University of Tennessee Health Science Center, United States of America

## Abstract

A key feature of the innate antiviral immune response is a rapid nonspecific response to virus infection largely mediated by the induction and extracellular secretion of type I interferons (IFNs) that restrict virus replication. Cytoplasmic sensors such as RIG-I recognize viral RNA and trigger antiviral signaling pathways that upregulate IFN transcription. However, it remains largely unknown how antiviral signaling is negatively regulated to maintain homeostasis after the elimination of virus. In this report, we have identified the RING domain-containing protein RING finger 11 (RNF11) as a novel negative regulator of innate antiviral signaling. Overexpression of RNF11 downregulated IFN-β expression and enhanced viral replication whereas siRNA-mediated knockdown of RNF11 suppressed viral replication. RNF11 interacted with the noncanonical IKK kinases TBK1/IKKi and attenuated their Lys63-linked polyubiquitination by blocking interactions with the E3 ligase TRAF3. The inhibitory function of RNF11 was dependent on the ubiquitin-binding adaptor molecule TAX1BP1 which was required for RNF11 to target TBK1/IKKi. Collectively, these results indicate that RNF11 functions together with TAX1BP1 to restrict antiviral signaling and IFN-β production.

## Introduction

Innate immunity provides a rapid nonspecific antiviral state that impedes viral infection and spread [1]. This generally involves the rapid detection and clearance of the virus via the production of interferon (IFN) stimulated genes and later the activation of the adaptive immune response [2]. Upon virus entry and replication, double stranded RNA (dsRNA) is produced and serves as a pathogen associated molecular pattern (PAMP) that is detected by host pattern recognition receptors (PRRs). A hallmark feature of innate antiviral immunity is a rapid induction of type I IFNs (IFN-α and IFN-β) which are secreted from infected cells and engage the IFN-αβ receptor on neighboring cells. IFN signaling then triggers the induction of interferon stimulated genes (ISGs) that together coordinate an antiviral response to suppress viral replication and infection [3].

There are two major classes of PRRs, Toll-like receptors (TLRs) and the retinoic acid-inducible-gene-I (RIG-I)-like helicase receptors (RLRs) [4]. A major difference between these PRRs is their cellular localization with TLRs localized within the plasma membrane or within intracellular vesicles whereas RLRs are cytoplasmic sensors for viral infection [5]. Two of the best characterized RLRs are RIG-I and melanoma differentiation-associated gene 5 (MDA5) that each recognize distinct viral PAMPs [6]. RIG-I recognizes uncapped 5′-triphosphate RNA produced by RNA viruses such as influenza [7], [8]. Conversely, MDA5 is critical for picornavirus detection and also senses dsRNA including the synthetic dsRNA analog poly(I:C) [9]. Upon virus infection, RIG-I undergoes Lys63-linked polyubiquitination by the E3 ligase TRIM25 [10] to trigger the binding with the mitochondrial adaptor IPS-1 (also known as VISA, Cardif, or MAVS) via CARD-CARD interactions found in both proteins [11], [12]. IPS-1 then recruits the E3 ligase TRAF3 together with the noncanonical IκB kinases (IKKs) IKKi (also known as IKKε) and TANK-binding kinase 1 (TBK1) [13]–[17]. TBK1 and IKKi trigger the phosphorylation and subsequent dimerization of the transcription factor IRF3 leading to its nuclear translocation and recruitment to IFN stimulated response elements (ISREs) and the induction of type I IFN [18]. TBK1 and IKKi are conjugated with Lys63-linked polyubiquitin chains during virus infection as a mechanism to promote IRF3 activation [19]–[21]. TBK1/IKKi Lys63-linked polyubiquitination appears to be a critical event for IFN production since several negative regulators of the RIG-I pathway target TBK1/IKKi polyubiquitination [19], [22], [23].

RING finger protein 11 (RNF11) is an evolutionarily conserved 154 amino acid protein that was originally found to be overexpressed in breast tumors [24]. RNF11 contains an amino (N)-terminal myristoylation domain and a PPXY (where P = proline, X = any amino acid and Y = tyrosine) motif that mediates interactions with Homologous to the E6-AP Carboxyl Terminus (HECT) E3 ligases Itch, Smurf1, and Smurf2 via WW domains [25], [26]. RNF11 also contains a really interesting gene (RING) domain at its carboxyl (C)-terminus [27] and interacts with the E2 ubiquitin conjugating enzymes UbcH5a, b and c [28] indicating that RNF11 may function as an E3 ubiquitin ligase. Indeed, RNF11 has been implicated as a regulator of transforming growth factor beta (TGF-β) signaling pathways by modulating the ubiquitination and proteolysis of receptors and signaling intermediates [27]. RNF11 has been proposed to augment TGF-β signaling by counteracting the ubiquitin-mediated proteolysis of Smad2 and the TGF-β receptor by the E3 ligase Smurf2 [29]. A large scale yeast two-hybrid screen using RNF11 as bait was conducted to identify novel regulators of the TGF-β pathway yielding A20, TAX1BP1, and Itch [30], all of which are important negative regulators of the NF-κB and antiviral signaling pathways. A20 is a ubiquitin-editing enzyme that is critical for the inhibition of NF-κB signaling and is regulated by the ubiquitin-binding adaptor molecule TAX1BP1 and the HECT domain E3 ligase Itch [31]. A20, TAX1BP1, Itch and RNF11 form a cytokine-inducible ubiquitin-editing complex that downregulates RIP1 and TRAF6 Lys63-linked polyubiquitination and TNF and IL-1-mediated NF-κB activation [32].

The RIG-I/MDA5 pathway is tightly regulated by inhibitory proteins to prevent deleterious overproduction of type I IFNs that may contribute to the genesis of autoimmune diseases such as systemic lupus erythematosus (SLE) [33]. Our previous studies as well as others have found that numerous inhibitors of the NF-κB pathway also function as inhibitors of antiviral signaling including A20, TAX1BP1, Itch and ABIN1 suggesting that these proteins may form an antiviral ubiquitin-editing complex analogous to what occurs during inflammatory signaling [22], [23], [34], [35]. In support of this model, A20, TAX1BP1 and ABIN1 cooperate and are dependent on each other to target TBK1/IKKi for inactivation to attenuate antiviral signaling [22], [23]. Although RNF11 is a key component of the anti-inflammatory A20 ubiquitin-editing complex [36], its role in antiviral signaling is unknown. Here we report that RNF11 is a novel inhibitor of the RIG-I/MDA5 pathway by blocking Lys63-linked polyubiquitination of TBK1/IKKi. Furthermore, RNF11 requires TAX1BP1 to interact with TBK1/IKKi and inhibit antiviral signaling. Together, this study suggests that RNF11 is a novel inhibitor of antiviral signaling that cooperates with TAX1BP1 to restrict IFN production during virus infection.

## Results

### RNF11 inhibits antiviral signaling and virus-induced IFN-β production

**Figure 1:**
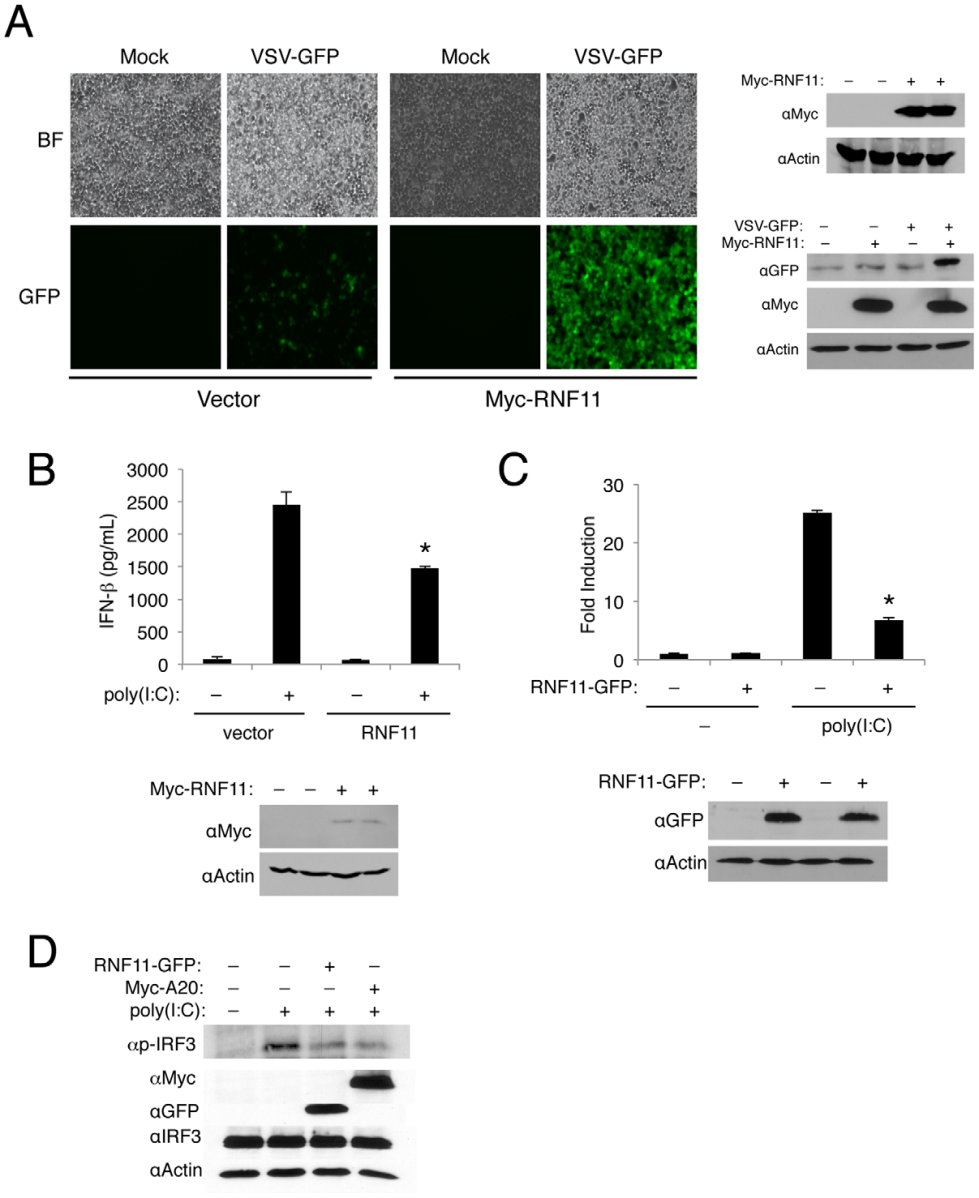
RNF11 is a negative regulator of virus-induced IFN-β production. (A) Micrographs of 293T cells transfected with either empty vector or Myc-RNF11 and then infected with VSV-GFP (MOI of 0.1) 24 h later. Pictures were taken 24 h post-infection. Immunoblotting was conducted with protein lysates using anti-Myc, anti-GFP and anti-Actin (right panel). (B) MEFs were transfected with either empty vector or Myc-RNF11 and were transfected again with poly(I:C) (15 μg) 24 h later. An IFN-β ELISA was performed 16 h later using supernatants. Immunoblotting was conducted with anti-Myc and anti-Actin. (C) 293T cells were transfected with an IFN-β luciferase reporter (200 ng), pRL-tk (20 ng), empty vector (1 µg) or RNF11-GFP (1 µg). Cells were transfected 24 h later with poly(I:C) (15 µg) and dual luciferase assays were performed after 16 h. Immunoblotting was conducted with protein lysates using anti-GFP and anti-Actin. (D) 293T cells were transfected with RNF11-GFP (1 μg) and Myc-A20 (1 μg) and then transfected 24 h later with poly(I:C) (20 µg). Immunoblotting was performed with anti-p-IRF3, anti-Myc, anti-GFP, anti-IRF3 and anti-Actin.

RNF11 is a key negative regulator of NF-κB by functioning as a subunit of the A20 ubiquitin-editing complex [36]. Given that the NF-κB inhibitors A20, TAX1BP1, and ABIN1 inhibit antiviral signaling and interact with RNF11 [22], [23], [34], we hypothesized that RNF11 may also block antiviral signaling. We first examined the effect of overexpressed RNF11 on the replication of the negative-sense RNA virus vesicular stomatitis virus (VSV) expressing the green fluorescent protein (GFP) in 293T cells using fluorescence microscopy. Replication of VSV-GFP was evident from cells expressing GFP and in the presence of Myc-RNF11 the replication of VSV-GFP was greatly enhanced (Fig. 1A). Immunoblotting also confirmed increased expression of GFP in the presence of Myc-RNF11 (Fig. 1A). Similar results were obtained in murine embryonic fibroblasts (MEFs) infected with VSV-GFP ( Fig. S1). Therefore, overexpression of RNF11 leads to more robust viral replication in multiple cell types. Conversely, depletion of RNF11 with short interfering RNA (siRNA) inhibited the replication of VSV-GFP (Fig. S2). To determine if RNF11 was enhancing viral replication by suppressing the production of type I IFN, we next examined the effect of RNF11 on IFN-β production by ELISA. MEFs were transfected with the dsRNA mimetic poly(I:C) together with empty vector or Myc-RNF11. Overexpression of RNF11 significantly blocked poly(I:C)-induced production of IFN-β (Fig. 1B). To gain more insight into how RNF11 was inhibiting the production of IFN-β, we conducted a luciferase reporter assay to monitor IFN-β promoter activation. RNF11 significantly inhibited poly(I:C)-induced activation of the IFN-β promoter (Fig. 1C). Taken together, RNF11 appears to be a novel negative regulator of the RIG-I/MDA5 antiviral pathway.

Upon virus infection the transcription factor IRF3 is phosphorylated within its C-terminal domain by TBK1/IKKi to trigger its dimerization and nuclear localization [18]. Phosphorylated IRF3 can be detected with a phospho-specific antibody that recognizes IRF3 phosphorylated on Ser-386. Therefore, we next examined if RNF11 blocked the phosphorylation of IRF3. As expected, poly(I:C) transfection induced IRF3 phosphorylation that was attenuated by A20 as previously described [34] (Fig. 1D). RNF11 also inhibited the phosphorylation of IRF3 to a similar extent as A20 (Fig. 1D). Therefore, RNF11 blocks the phosphorylation of IRF3 as part of its mechanism to inhibit virus-induced IFN-β.

Previous studies have demonstrated that RNF11 contains an N-terminal myristoylation motif important for membrane targeting [25] and a PPXY motif that mediates interactions with the HECT domain E3 ligases Itch and Smurf2 [26]. Site-directed mutagenesis of RNF11 was performed to determine potential contributions of each of these domains for the inhibition of antiviral signaling. Mutations were introduced in RNF11 fused with GFP at its C-terminus (RNF11-GFP) to allow for a free N-terminal end necessary for membrane localization of RNF11 [25]. The glycine residue critical for myristoylation was mutated to alanine (G2A) and the tyrosine within the PPXY motif was also mutated to alanine (Y40A). Both the myristoylation and PPXY motifs were dispensable for the inhibition of poly(I:C)-induced activation of the IFN-β reporter (Fig. S3). In fact, we consistently observed stronger inhibition with the RNF11 myristoylation mutant (Fig. S3) suggesting that RNF11 inhibits antiviral signaling outside of membrane compartments. We also generated an RNF11 mutant lacking the RING domain (RNF11ΔRING) to ascertain the role of RNF11 E3 ligase activity for inhibition of antiviral signaling. Surprisingly, RNF11 lacking the RING domain was still able to inhibit poly(I:C)-induced activation of the IFN-β reporter (Fig. S3). Collectively, these results indicate that RNF11 inhibits antiviral signaling in the absence of its E3 ligase activity, membrane targeting and interaction with HECT domain E3 ligases.

### RNF11 blocks antiviral signaling at the level of TBK1/IKKi

**Figure 2:**
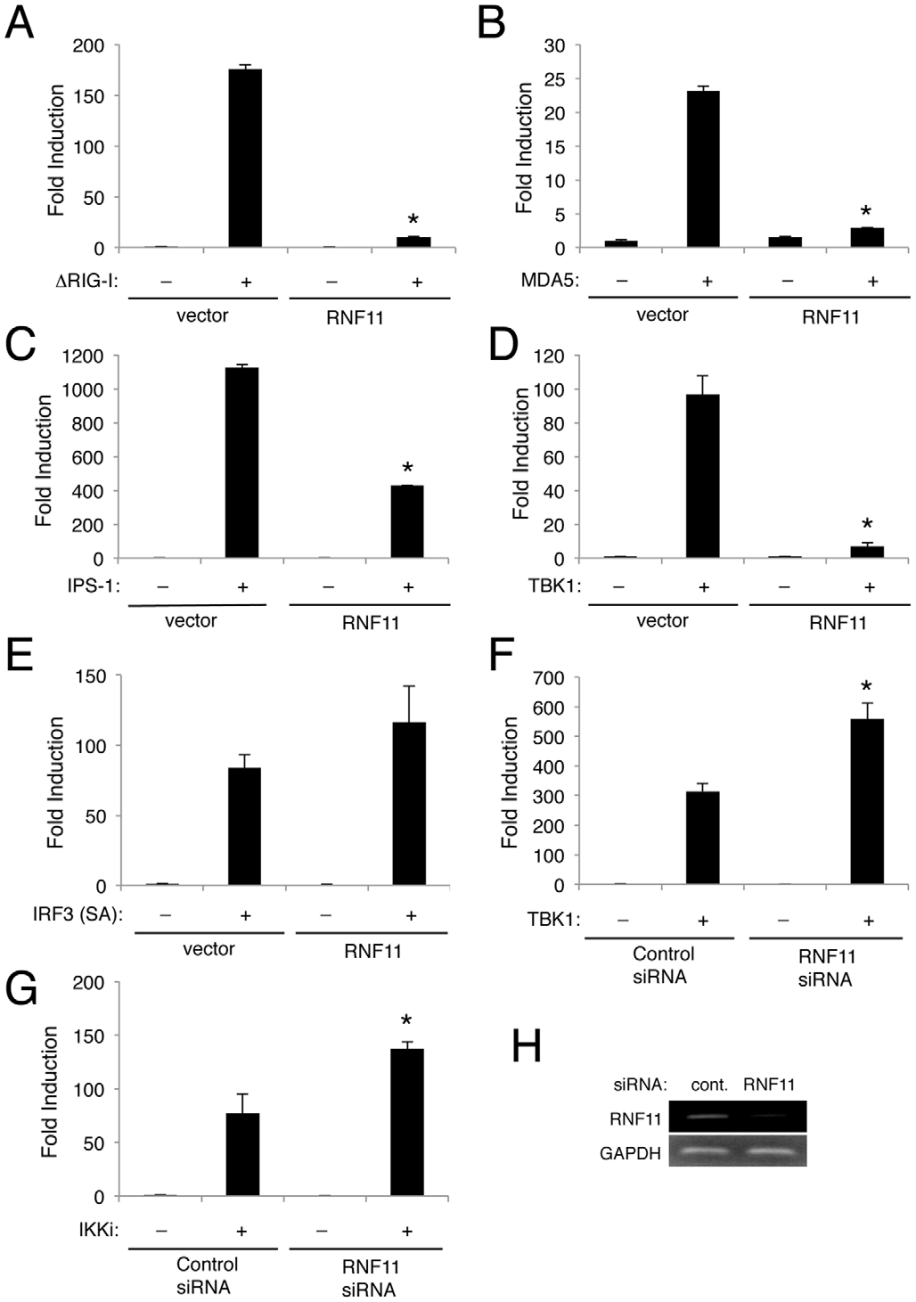
RNF11 inhibits IFN-β production at the level of TBK1/IKKi. (A–E) 293T cells were transfected with IFN-β luciferase reporter (200 ng), pRL-tk (20 ng), empty vector (1 μg), RNF11-GFP (1 μg) and either 0.5 μg of ΔRIG-I (A), MDA5 (B), IPS-1 (C), TBK1 (D) or IRF3-SA (E). Dual luciferase assays were performed with protein lysates 24 h later. (F, G) 293T cells were transfected with either control scrambled or RNF11 siRNA (60 pmol). After 24 h, cells were transfected with IFN-β luciferase reporter (200 ng), pRL-tk (20 ng), and either Flag-TBK1 or Flag-IKKi (0.5 μg) and dual luciferase assays were performed 24 h later. (H) 293T cells were transfected with either control scrambled or RNF11 siRNA (60 pmol). After 48 h, RT-PCR was performed to detect RNF11 and Actin transcripts. *, p<0.05. *Error bars, S.D.*

In order to determine the target(s) of RNF11 in the negative regulation of the antiviral signaling, we next overexpressed key signaling molecules in the RIG-I/MDA5 pathway. Overexpression of the RIG-I CARD domain (ΔRIG-I) strongly activated the IFN-β reporter but was significantly inhibited by RNF11 (Fig. 2A). Similarly, RNF11 also blocked MDA5-induced activation of the IFN-β promoter (Fig. 2B). IPS-1 strongly induced IFN-β promoter activation that was also inhibited by RNF11 (Fig 2C). Although RNF11 potently blocked IFN-β promoter activation by TBK1 overexpression, it was unable to inhibit a constitutively active form of IRF3 (IRF SA) (Figs. 2D, E). These data collectively suggest that RNF11 inhibits antiviral signaling upstream of IRF3 at the level of TBK1. Consistent with this notion, siRNA-mediated knockdown of RNF11 significantly enhanced IFN-β promoter activation by either TBK1 or IKKi (Fig. 2F, G). Efficient knockdown of RNF11 was confirmed by RT-PCR (Fig. 2H). Therefore, RNF11 appears to target both TBK1 and IKKi for inhibition of the RIG-I/MDA5 pathway.

### RNF11 interacts with TBK1/IKKi and blocks their Lys63-linked polyubiquitination

**Figure 3:**
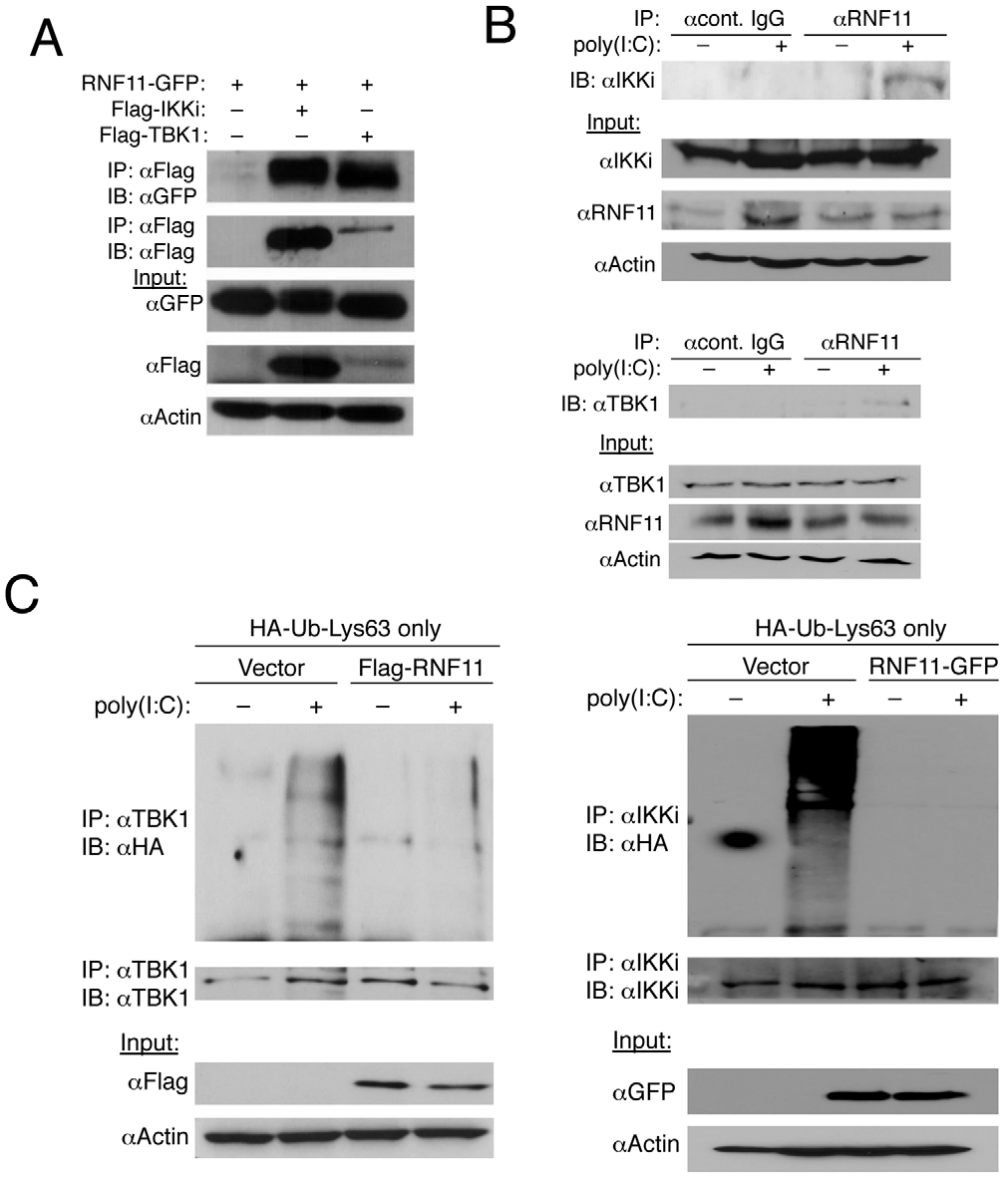
RNF11 interacts with TBK1/IKKi and blocks their Lys63-linked polyubiquitination. (A) 293T cells were transfected with 1 µg of RNF11-GFP, Flag-IKKi and Flag-TBK1. Co-IPs were conducted using anti-Flag for IP followed by immunoblotting with anti-GFP and anti-Flag. Immunoblotting was performed with lysates using anti-GFP, anti-Flag and anti-Actin. (B) 293T cells were transfected with poly(I:C) (20 μg) and co-IPs were performed with anti-RNF11 or isotype control IgG followed by immunoblotting with anti-IKKi (top panel) or anti-TBK1 (lower panel). Immunoblots were also performed with lysates using anti-IKKi, anti-RNF11, anti-TBK1 and anti-Actin. (C) 293T cells were transfected with empty vector, Flag-RNF11 or RNF11-GFP (1 µg) and HA-Ub-Lys63-only (500 ng). Cells were transfected again 24 h later with poly(I:C) (20 μg) and co-IPs were conducted the next day using anti-TBK1 (left panel) or anti-IKKi (right panel) followed by immunoblotting with anti-HA, anti-TBK1 (left panel) and anti-IKKi (right panel). Immunoblotting was performed with lysates with anti-Flag, anti-GFP and anti-Actin.

Next, we sought to determine how RNF11 inhibits TBK1/IKKi activation to dampen antiviral signaling and IFN-β production. Co-immunoprecipitation (co-IP) experiments were conducted to determine if RNF11 interacts with TBK1 and IKKi. Under overexpression conditions, RNF11 indeed interacted with both TBK1 and IKKi (Fig. 3A). However, interactions between endogenous RNF11 and TBK1/IKKi were only observed when cells were transfected with poly(I:C) (Fig. 3B). Therefore, RNF11 is likely recruited to both TBK1 and IKKi only when they are activated in response to virus infection.

TBK1 and IKKi both undergo Lys63-linked polyubiquitination following virus infection, presumably to recruit a signaling complex containing ubiquitin binding proteins that activate IRF3 [19], [20]. Our previous studies as well as others have demonstrated that A20, TAX1BP1, ABIN1 and CYLD inhibit TBK1/IKKi by antagonizing their Lys63-linked polyubiquitation [19], [22], [23]. Therefore, we next examined if RNF11 blocks the Lys63-linked polyubiquitination of TBK1/IKKi. For this purpose, we used an HA-ubiquitin (Ub) plasmid with all lysines mutated to arginines except for Lys63 (Lys63 only) to facilitate selective Lys63-linked polyubiquitination [37]. Endogenous TBK1 and IKKi were conjugated with Lys63-linked polyubiquitin chains only upon transfection with poly(I:C) as expected (Fig. 3C). However, ectopic expression of RNF11 completely blocked the Lys63-linked polyubiquitination of both TBK1 and IKKi (Fig. 3C). Collectively, these data suggest that RNF11 is recruited to TBK1 and IKKi upon virus infection and attenuates their Lys63-linked polyubiquitination to block IFN-β production.

### RNF11 inhibits the interactions between TRAF3 and IKKi

**Figure 4:**
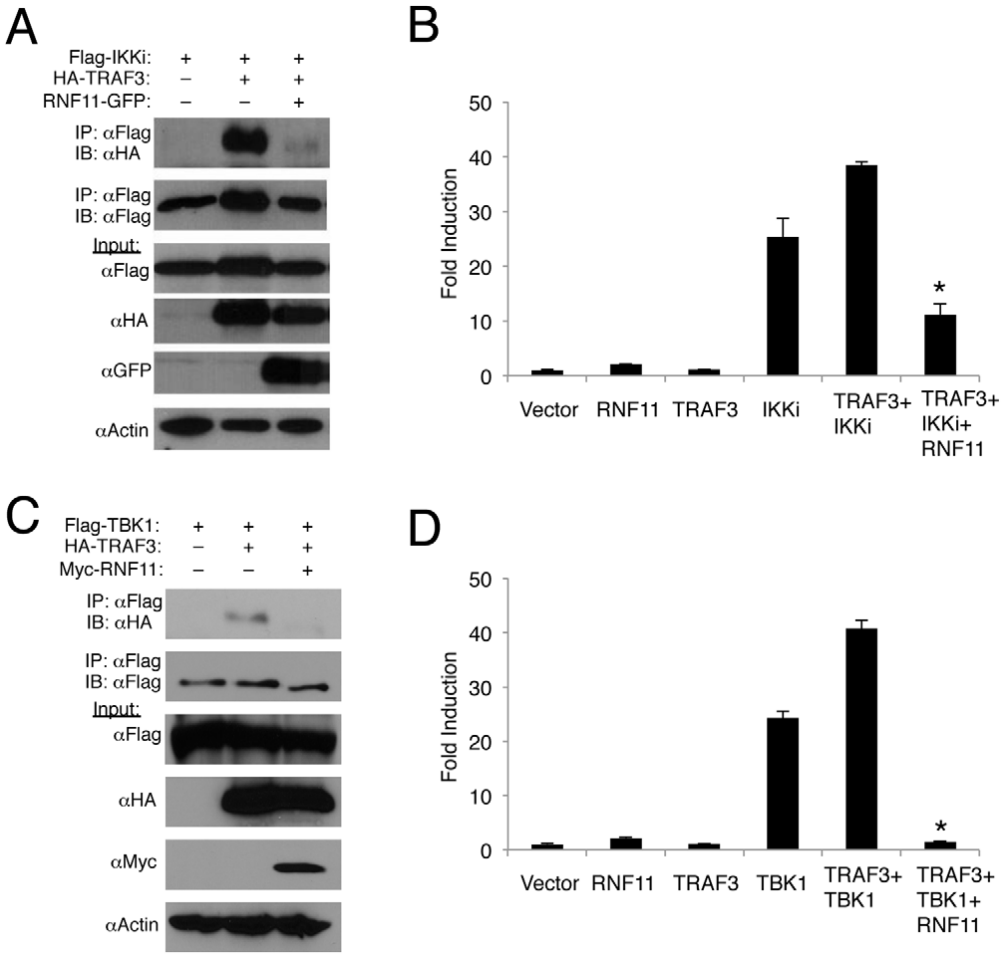
RNF11 disrupts the interaction between TRAF3 and TBK1/IKKi. (A, C) 293T cells were transfected with 1 μg of either HA-TRAF3, Flag-IKKi, Flag-TBK1 or RNF11-GFP. Co-IPs were conducted using anti-Flag followed by immunoblotting with anti-HA and anti-Flag. Immunoblotting was performed with lysates using anti-Flag, anti-HA, anti-GFP and anti-Actin. (B, D) 293T cells were transfected with IFN-β luciferase reporter (200 ng), pRL-tk (20 ng), and 1 µg of RNF11, TRAF3, IKKi or TBK1. Dual luciferase assays were performed with protein lysates 24 h later. *, p<0.05. *Error bars, S.D.*

The E3 ligase TRAF3 plays a critical role in Toll-dependent and independent induction of IFN-β in response to virus infection [17]. Furthermore, we have demonstrated that TRAF3 synergizes with TBK1 to induce IFN-β expression and also plays an important role in the ubiquitination of IKKi [22]. We have also demonstrated that A20 and TAX1BP1 disrupt TRAF3 interactions with TBK1 and IKKi to attenuate their Lys63-linked polyubiquitination [22]. Therefore, we next examined if RNF11 could inhibit TRAF3 and TBK1/IKKi interactions. As expected, TRAF3 strongly interacted with TBK1/IKKi but this interaction was completely disrupted by RNF11 (Fig. 4A, C). Although TRAF3 alone was unable to induce IFN-β promoter activity as previously described [17], it synergized with TBK1/IKKi to enhance IFN-β promoter activation (Fig. 4B, D). Overexpression of RNF11 significantly blocked IFN-β promoter activation induced by TRAF3 together with TBK1/IKKi (Fig. 4B, D). Thus, RNF11 appears to inhibit Lys63-linked polyubiquitination of TBK1 and IKKi by disrupting interactions between TRAF3 and TBK1/IKKi.

### RNF11 is dependent on the adaptor molecule TAX1BP1 to inhibit antiviral signaling

**Figure 5:**
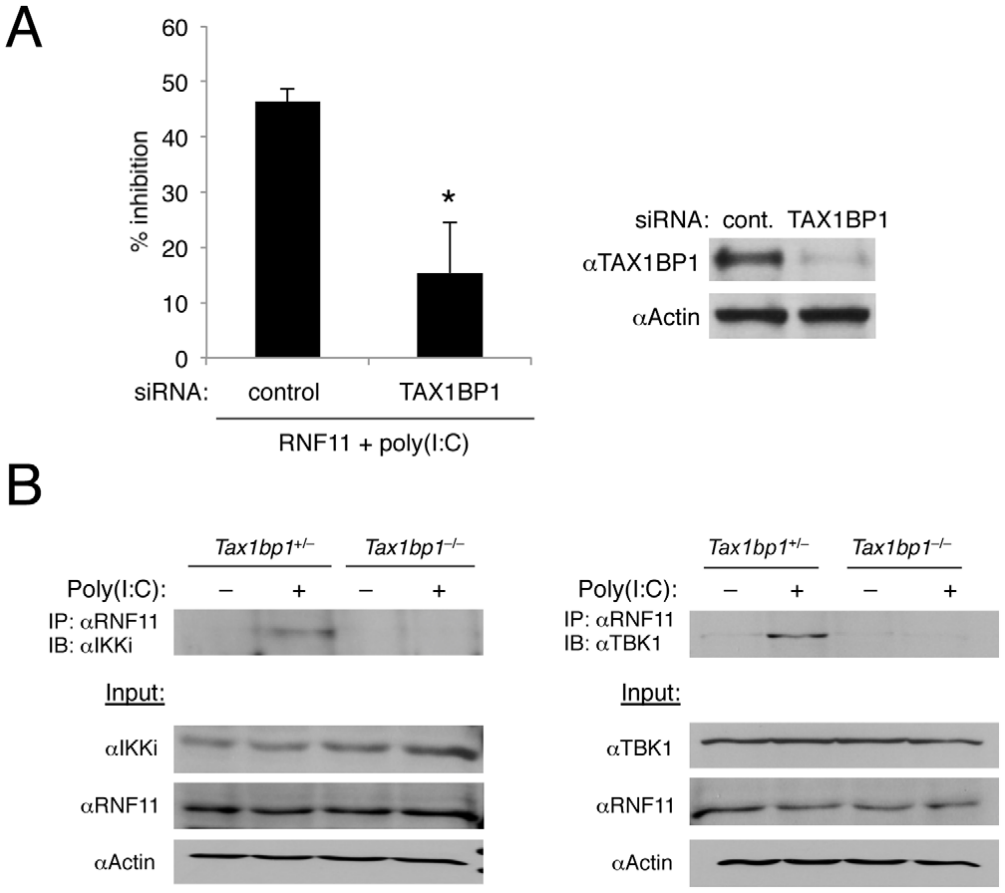
RNF11 requires TAX1BP1 to inhibit antiviral signaling. (A) 293T cells were transfected with IFN-β luciferase reporter (200 ng), pRL-tk (20 ng), RNF11 (1 µg) and either control scrambled or TAX1BP1 siRNA (60 pmol). After 24 h, cells were transfected with poly(I:C) (15 µg) and dual luciferase assays were performed 16 h later. The data is presented as percent inhibition by RNF11 of poly(I:C)-induced IFN-β promoter induction with either control scrambled or TAX1BP1 siRNA (left panel). Knockdown of TAX1BP1 was confirmed by immunoblotting using anti-TAX1BP1 and anti-Actin (right panel). (B) *Tax1bp1* ^+/–^ and *Tax1bp1* ^–/–^ MEFS were transfected with poly(I:C) (20 μg), and co-IPs were performed with anti-RNF11 followed by immunoblotting with anti-IKKi (left panel) or anti-TBK1 (right panel). Immunoblots were performed with lysates using anti-IKKi, anti-RNF11, anti-TBK1 and anti-Actin. *, p<0.05. *Error bars, S.D.*

Since RNF11 attenuated TBK1/IKKi ubiquitination and activation by a mechanism identical to what we have observed for A20 and TAX1BP1 [22], we considered the possibility that RNF11 was functioning together with A20 and TAX1BP1 in the context of an antiviral A20 ubiquitin-editing complex. Indeed, we previously demonstrated that RNF11 together with TAX1BP1, Itch and A20 formed a ubiquitin-editing complex that downregulated inflammatory signaling pathways [32], [36], [38]. First, we conducted TAX1BP1 siRNA knockdown experiments to determine if RNF11 required the TAX1BP1 adaptor molecule to inhibit antiviral signaling. Although RNF11 inhibited IFN-β promoter activation induced by poly(I:C), its inhibitory function was significantly diminished upon siRNA-mediated knockdown of TAX1BP1 (Fig. 5A). Knockdown of TAX1BP1 was confirmed by western blotting (Fig. 5A). If TAX1BP1 functions as an adaptor molecule for RNF11, we reasoned that RNF11 would be impaired in interacting with TBK1/IKKi in the absence of TAX1BP1. Indeed, RNF11 interacted with TBK1 and IKKi in a poly(I:C)-dependent manner in control MEFs, but this inducible interaction was abolished in *Tax1bp1* ^–/–^ MEFs (Fig. 5B). Thus, RNF11 is clearly dependent on TAX1BP1 to engage TBK1/IKKi for inactivation and the subsequent downregulation of IFN-β production.

## Discussion

In this study we have identified RNF11 as a novel negative regulator of the RIG-I/MDA5 pathway and virus-induced IFN-β production. RNF11 joins a growing list of negative regulators of the RIG-I/MDA5 pathway including CYLD, RNF125, NLRX1, SOCS1, TRIM21/Ro52, TRIM28, TRIM38, ISG56, optineurin, MIP-T3, Triad3a, NLRP4, RAUL, TRIP, NLRC5, DUBA, ITCH, A20, TAX1BP1 and ABIN1 [14], [19], [22], [23], [35], [39]–[54]. This large array of innate antiviral inhibitory proteins underscores the importance of homeostatic control of innate immune responses upon virus infection. Interestingly, the majority of these inhibitors function as either E3 ligases or deubiquitinases that target key effector molecules in the RIG-I/MDA5 pathway including RIG-I, IPS-1, TRAF3, TBK1/IKKi, IRF3 and IRF7. Ubiquitin therefore plays a critical role not only in the activation but also the termination of antiviral signaling.

RNF11 is a putative E3 ligase that we show here targets TBK1/IKKi Lys63-linked polyubiquitination to block virus-induced IFN-β production. Mechanistically, RNF11 cooperates with the adaptor molecule TAX1BP1 to target TBK1/IKKi for inhibition since RNF11 recruitment to TBK1/IKKi in response to poly(I:C) transfection was abolished in TAX1BP1-deficient MEFs. Our previous work identified RNF11 as a key negative regulator of NF-κB signaling in proinflammatory pathways by functioning as a component of the A20 ubiquitin-editing complex [36]. RNF11 likely plays a similar role in antiviral signaling since we have shown that A20, TAX1BP1, ABIN1 and RNF11 are all interacting proteins that inhibit TBK1/IKKi polyubiquitination [22], [23]. Optineurin may also be a component of the A20 antiviral complex since it interacts with TRAF3 and TBK1 and inhibits antiviral signaling at the level of TBK1 [48]. Optineurin was also identified as an RNF11 interacting protein in a yeast two-hybrid screen [30]. Since the deubiquitinase (DUB) activity of A20 is not required to block virus-induced IFN-β production [22], the A20 antiviral ubiquitin-editing complex likely disrupts interactions between TBK1/IKKi and upstream molecules such as TRAF3 to attenuate the Lys63-linked polyubiquitination of TBK1/IKKi. Since multiple E3 ligases have been proposed to conjugate ubiquitin onto TBK1 during virus infection including TRAF3, Mind bomb and Nrdp1 [20]–[22], it is possible that RNF11 (and the A20 complex) also blocks TBK1/IKKi interactions with Mind bomb and Nrdp1.

RNF11 contains a myristoylation motif important for targeting RNF11 to membrane compartments such as endosomes and mutation of this motif results in a diffuse cytoplasmic staining of RNF11 [25]. However, membrane targeting of RNF11 is dispensable for inhibition of antiviral signaling (Fig. S3), suggesting that RNF11 inhibits antiviral signaling outside of membrane compartments. We previously demonstrated that the PPXY motif in RNF11 is important to suppress NF-κB signaling, likely due to binding with the E3 ligase Itch [36]. Nevertheless, the PPXY motif is not required for RNF11 to inhibit antiviral signaling (Fig. S3), indicating that interactions with Itch or other WW domain containing proteins may not be necessary for its inhibitory function in antiviral signaling. Although Itch has been described as a negative regulator of the RIG-I/MDA5 pathway, it was shown to inhibit upstream of TBK1/IKKi by triggering the degradation of IPS-1 [35]. Surprisingly, an RNF11 mutant lacking the RING domain still inhibited the IFN-β promoter (Fig. S3). RNF11 therefore may function as an adaptor molecule or scaffold protein to inhibit antiviral signaling. Taken together, RNF11 inhibits antiviral signaling in the absence of its membrane targeting domain, PPXY motif and RING domain.

It is currently unclear what the precise role is for each of the members of the A20 antiviral complex (A20, TAX1BP1, ABIN1 and RNF11) in TBK1/IKKi inhibition. Although disruption of TRAF3 and TBK1/IKKi interactions appears to be an important part of the mechanism, the contributions of each of the proteins in the A20 antiviral complex needs to be determined in future studies. Both TAX1BP1 and ABIN1 contain ubiquitin-binding domains (UBDs) that interact with either Lys63-linked or linear polyubiquitin chains [55]–[58]. Therefore, TAX1BP1 and ABIN1 likely sense activated and hence ubiquitinated TBK1/IKKi to target them for inactivation by recruiting A20 and RNF11. Therefore, A20 and RNF11 may play critical roles in the disruption of protein-protein interactions to attenuate TBK1/IKKi ubiquitination. It is also unclear what regulates the interactions between A20, TAX1BP1, ABIN1 and RNF11 during virus infection to assemble the A20 antiviral complex. We have recently demonstrated that IKKα-induced phosphorylation of TAX1BP1 is the key event that nucleates the anti-inflammatory A20 ubiquitin-editing complex [32]. A similar post-translational modification of TAX1BP1 or one of the other subunits may also regulate assembly of the A20 antiviral complex.

A recent study described a splice site variant in the *Rnf11* gene in Belgian Blue Cattle that resulted in growth retardation and premature lethality due to uncontrolled inflammation in the respiratory tract [59]. Although the underlying trigger of the inflammatory response in these animals is unclear, this study provides *in vivo* evidence for an important role of RNF11 in the resolution of inflammation. Since RNF11 also blocks type I IFN production, these animals may be more resistant to virus infection. Ongoing studies with RNF11-deficient mice will undoubtedly provide new insight into the role of RNF11 in the negative regulation of antiviral signaling and inflammation.

## Materials and Methods

### Antibodies and siRNAs

Flag M2 and RNF11 antibodies were purchased from Sigma. GFP, HA, IRF3, and TRAF3 antibodies were purchased from Santa Cruz Biotechnologies, Inc. Myc antibody was purchased from Millipore. Phospho-IRF3, TBK1, TAX1BP1 and β-actin antibodies were purchased from Abcam. IKKi antibody was purchased from Imgenex. Control scrambled, SmartPOOL TAX1BP1, and RNF11 siRNAs were purchased from Dharmacon/Thermo Scientific. Poly(I:C) was purchased from Invivogen.

### Plasmids and Mutagenesis

Plasmids encoding A20, ΔRIG-I, IPS-1, RNF11, MDA5, TBK1, IKKi, IRF3 (SA), TRAF3, HA-Ub-Lys63-only, and the IFN-β luciferase reporter have been described previously [22], [23], [36]. The RNF11 cDNA was subcloned into the pEGFP-N1 vector (Clontech) using Xho1 and Hind III to generate RNF11-GFP. The RNF11ΔRING mutant (1-98) was also fused with GFP using the same strategy. Site-directed mutagenesis of RNF11 was performed using the QuikChange site-directed mutagenesis kit (Stratagene) according to the manufacturer's instructions.

### ELISA

ELISAs for mouse IFN-β were performed using supernatants from MEFs [32] infected with virus. Values are expressed as pg/mL ± S.D. as calculated from a standard curve derived from recombinant IFN-β provided in the ELISA kit (PBL Interferon Source).

### Cell Culture, Transfections, and Reporter Assays

293T cells were purchased from ATCC. *Tax1bp1* ^+/–^ and *Tax1bp1* ^–/–^ MEFs were described previously [60]. 293T and MEFs were cultured in DMEM supplemented with 10% Fetal Bovine Serum and 1% penicillin/streptomycin. FuGENE 6 and FuGENE HD (Roche Applied Science) were used to transfect 293T cells or MEFs, respectively. siRNAs (60 pmol) were transfected using Lipofectamine 2000 (Invitrogen). Reporter assays were performed 24 h post DNA transfection unless otherwise indicated using a dual luciferase assay kit (Promega). Results for firefly luciferase activity were normalized to *Renilla* luciferase activity. Data are expressed as mean-fold increase ± S.D. relative to the control from a representative experiment performed three times in duplicate or triplicate. An asterisk indicates a *p* value of <0.05 as determined by Student's *t* test.

### Immunoblotting, Co-IPs, and Ubiquitination Assays

Whole cell lysates were produced by lysing cells in RIPA buffer (50 mM Tris-Cl, pH 7.4, 150 mM NaCl, 1% Nonidet P-40, 0.25% sodium deoxycholate, 1 mM PMSF, 1x Roche complete mini protease inhibitor mixture) on ice, followed by centrifugation. Cell lysates were separated by SDS-PAGE, transferred to nitrocellulose membranes, and subjected to immunoblotting. For co-IPs, lysates were diluted 1:1 in RIPA buffer and precleared with protein A-agarose beads for 60 min at 4°C. Precleared lysates were further incubated at 4°C overnight with the indicated antibodies (1–3 µL) and protein A-agarose beads. Immunoprecipitates were washed three times with RIPA buffer followed by elution of bound proteins with 2x Laemmli sample. For ubiquitination assays, an extra wash was performed using RIPA buffer supplemented with 1M Urea.

### Virus Infections

293T cells were infected with VSV encoding GFP (VSV-GFP) [61] at a multiplicity of infection (MOI) of 0.1 for 24 h.

### RT-PCR

Reverse Transcription-PCR (RT-PCR) was performed as described previously [62]. The RNF11 forward primer sequence was 5′- ATG GGG AAC TGC CTC AAA TCC -3′. The RNF11 reverse primer sequence was: 5′- TCA ATT AGT CTC ATA GGA TGA AAG -3′. The GAPDH forward primer sequence was 5′- CCA CAG TCC ATG CCA TCA C -3′. The GAPDH Reverse primer sequence was: 5′- GCT TCA CCA CCT TCT TGA TG -3′.

## Supporting Information

Figure S1 **Overexpression of RNF11 enhances virus replication in MEFs.** Micrographs of MEFs transfected with either empty vector or Myc-RNF11 and then infected with VSV-GFP (MOI of 0.1) 24 h later. Pictures were taken 24 h post-infection.
(TIF)

Figure S2 **Knockdown of RNF11 with siRNA inhibits virus replication.** Micrographs of 293T cells transfected with either control siRNA or RNF11 siRNA and then infected with VSV-GFP (MOI of 0.1) 24 h later. Pictures were taken 24 h post-infection. RT-PCR was conducted for RNF11 and GAPDH (lower panel).
(TIF)

Figure S3 **The membrane targeting domain, PPXY motif and RING domain are dispensable for RNF11 to inhibit antiviral signaling.** (A, B) 293T cells were transfected with an IFN-β luciferase reporter (200 ng), pRL-tk (20 ng), and 1 μg of either RNF11-GFP, RNF11-GFP G2A, RNF11-GFP Y40A or RNF11-GFP ΔRING. Cells were transfected 24 h later with poly(I:C) (15 µg) and dual luciferase assays were performed with protein lysates after 16 h. Immunoblotting was conducted with protein lysates using anti-GFP and anti-Actin.
(TIF)
