# Supplementary figures and images for: RING Finger Protein 11 Targets TBK1/IKKi Kinases to Inhibit Antiviral Signaling
Source: PLoS One. 2013 Jan 7;8(1):e53717. doi: 10.1371/journal.pone.0053717 (PMC3538746; doi:10.1371/journal.pone.0053717)

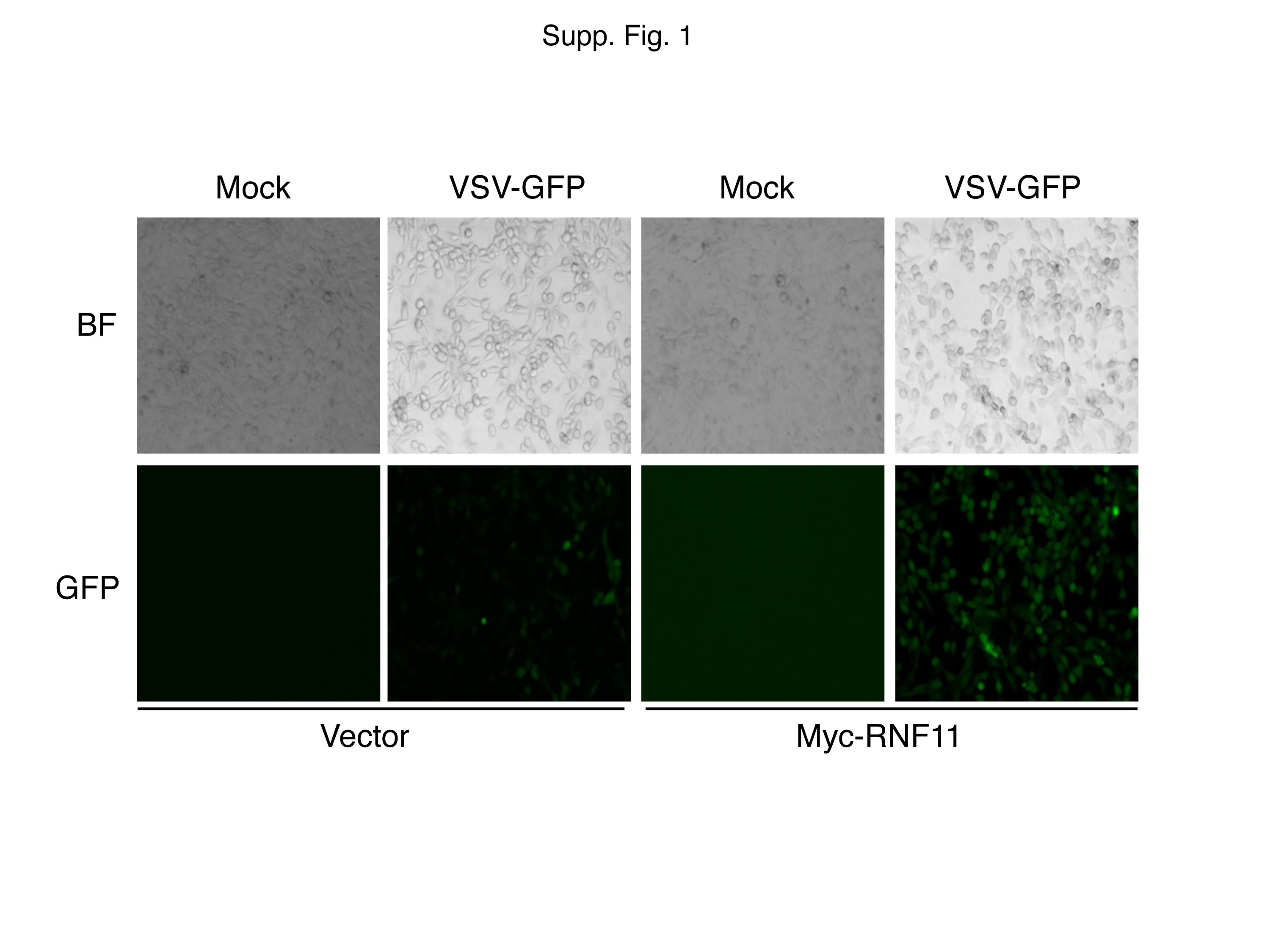

Supplement: Figure S1 — Overexpression of RNF11 enhances virus replication in MEFs. Micrographs of MEFs transfected with either empty vector or Myc-RNF11 and then infected with VSV-GFP (MOI of 0.1) 24 h later. Pictures were taken 24 h post-infection. (TIF) [file pone.0053717.s001.tif]

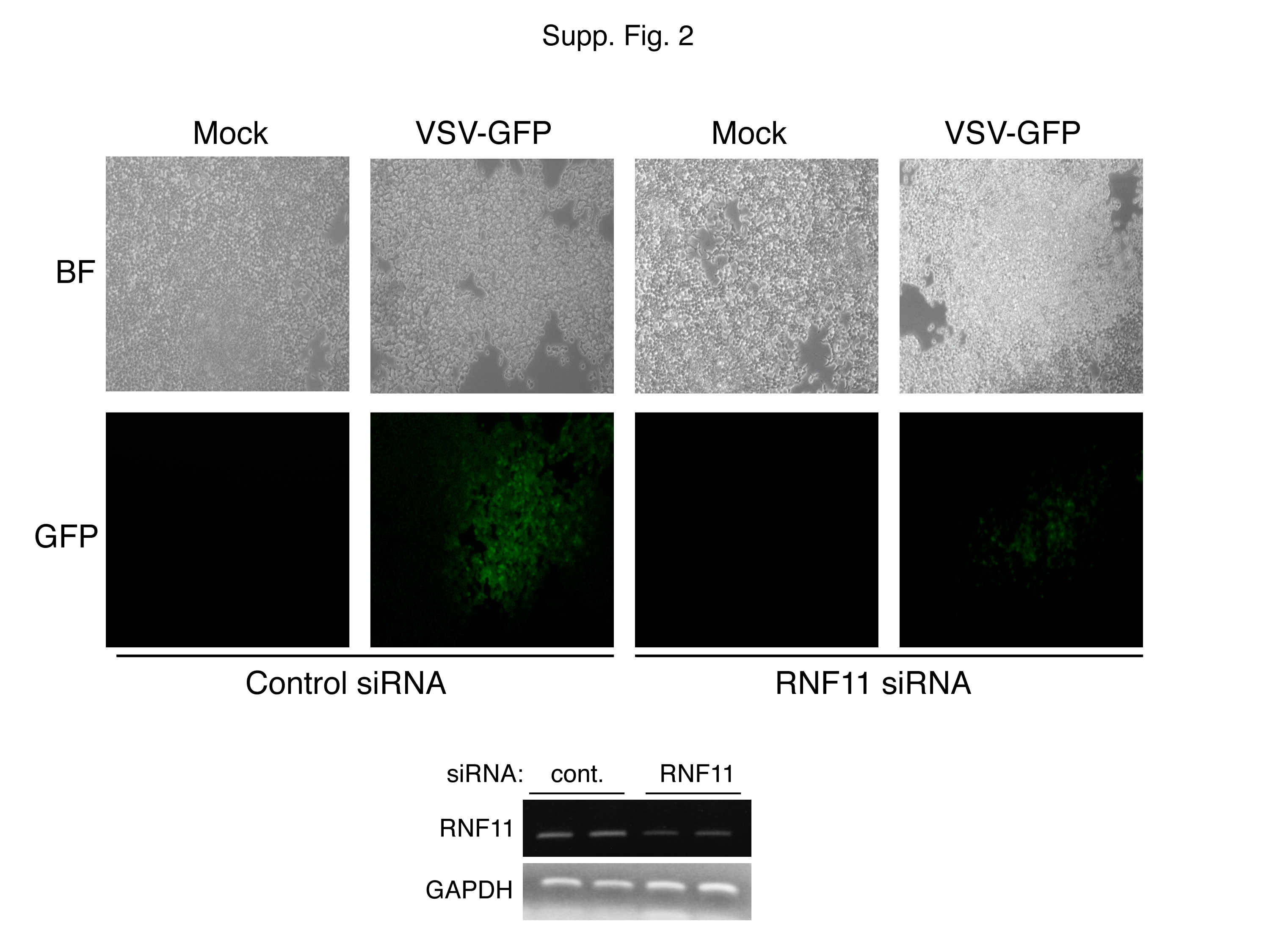

Supplement: Figure S2 — Knockdown of RNF11 with siRNA inhibits virus replication. Micrographs of 293T cells transfected with either control siRNA or RNF11 siRNA and then infected with VSV-GFP (MOI of 0.1) 24 h later. Pictures were taken 24 h post-infection. RT-PCR was conducted for RNF11 and GAPDH (lower panel). (TIF) [file pone.0053717.s002.tif]

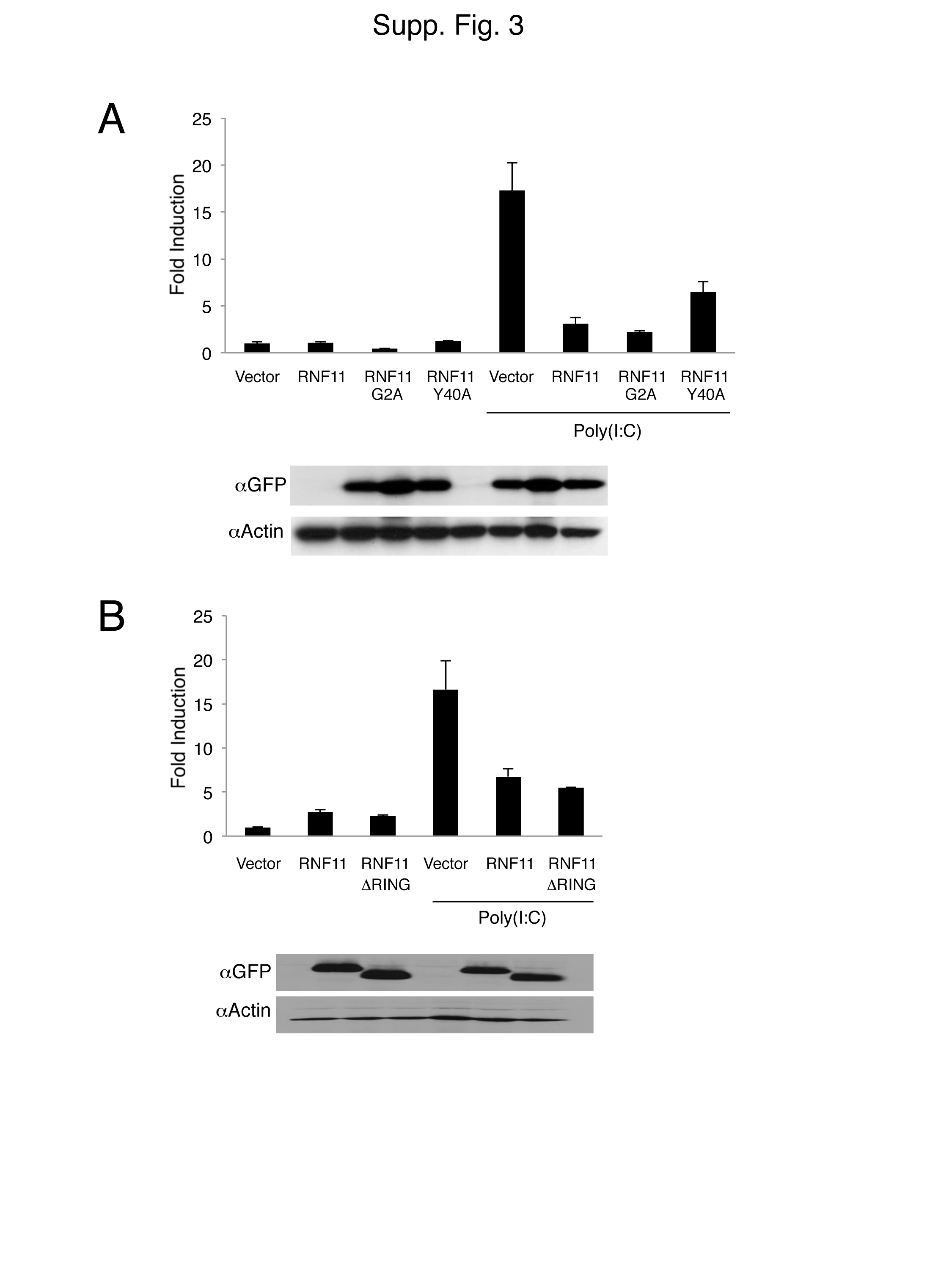

Supplement: Figure S3 — The membrane targeting domain, PPXY motif and RING domain are dispensable for RNF11 to inhibit antiviral signaling. (A, B) 293T cells were transfected with an IFN-β luciferase reporter (200 ng), pRL-tk (20 ng), and 1 μg of either RNF11-GFP, RNF11-GFP G2A, RNF11-GFP Y40A or RNF11-GFP ΔRING. Cells were transfected 24 h later with poly(I:C) (15 µg) and dual luciferase assays were performed with protein lysates after 16 h. Immunoblotting was conducted with protein lysates using anti-GFP and anti-Actin. (TIF) [file pone.0053717.s003.tif]
